# Supplementary material for: Causal Relationship between Aging and Anorexia Nervosa: A White-Matter-Microstructure-Mediated Mendelian Randomization Analysis
Source: Biomedicines. 2024 Aug 16;12(8):1874. doi: 10.3390/biomedicines12081874 (PMC11351342; doi:10.3390/biomedicines12081874)
Supplement: Supplementary file 1 [file biomedicines-12-01874-s001.zip › Supplementary Figure/Figure S4/Singe SNP plot of MR analysis for TL to ubm-b-1918.pdf]

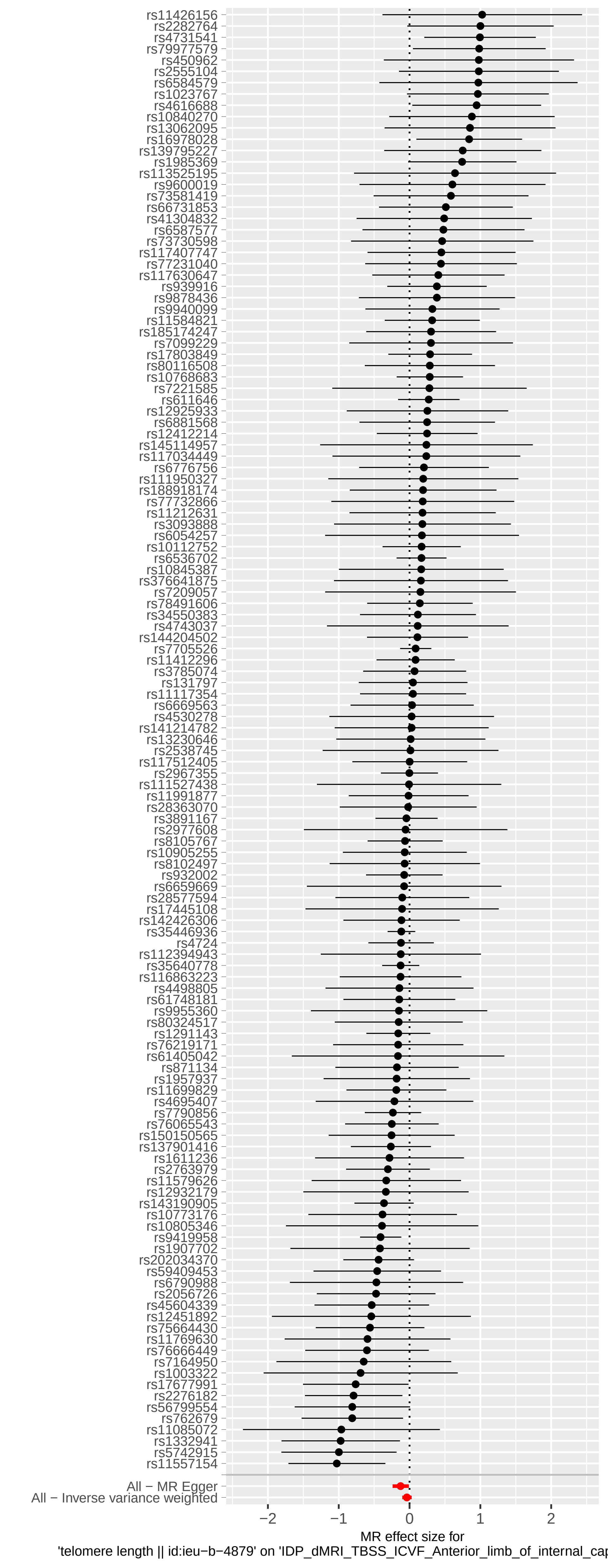
rs11426156
rs2282764
rs4731541
rs79977579
rs450962
rs2555104
rs6584579
rs1023767
rs4616688
rs10840270
rs13062095
rs16978028
rs139795227
rs1985369
rs113525195
rs9600019
rs73581419
rs66731853
rs41304832
rs6587577
rs73730598
rs117407747
rs77231040
rs117630647
rs939916
rs9878436
rs9940099
rs11584821
rs185174247
rs7099229
rs17803849
rs80116508
rs10768683
rs7221585
rs611646
rs12925933
rs6881568
rs12412214
rs145114957
rs117034449
rs6776756
rs111950327
rs188918174
rs77732866
rs11212631
rs3093888
rs6054257
rs10112752
rs6536702
rs10845387
rs376641875
rs7209057
rs78491606
rs34550383
rs4743037
rs144204502
rs7705526
rs11412296
rs3785074
rs131797
rs11117354
rs6669563
rs4530278
rs141214782
rs13230646
rs2538745
rs117512405
rs2967355
rs111527438
rs11991877
rs28363070
rs3891167
rs2977608
rs8105767
rs10905255
rs8102497
rs932002
rs6659669
rs28577594
rs17445108
rs142426306
rs35446936
rs4724
rs112394943
rs35640778
rs116863223
rs4498805
rs61748181
rs9955360
rs80324517
rs1291143
rs76219171
rs61405042
rs871134
rs1957937
rs11699829
rs4695407
rs7790856
rs76065543
rs150150565
rs137901416
rs1611236
rs2763979
rs11579626
rs12932179
rs143190905
rs10773176
rs10805346
rs9419958
rs1907702
rs202034370
rs59409453
rs6790988
rs2056726
rs45604339
rs12451892
rs75664430
rs11769630
rs76666449
rs7164950
rs1003322
rs17677991
rs2276182
rs56799554
rs762679
rs11085072
rs1332941
rs5742915
rs11557154
All − MR Egger
All − Inverse variance weighted
−2
−1
0
1
2
MR effect size for
'telomere length || id:ieu−b−4879' on 'IDP_dMRI_TBSS_ICVF_Anterior_limb_of_internal_cap
